# Discordance between the triglyceride glucose index and fasting plasma glucose or HbA1C in patients with acute coronary syndrome undergoing percutaneous coronary intervention predicts cardiovascular events: a cohort study from China

**DOI:** 10.1186/s12933-020-01091-8

**Published:** 2020-07-23

**Authors:** Chengping Hu, Jianwei Zhang, Jinxing Liu, Yan Liu, Ang Gao, Yong Zhu, Yingxin Zhao

**Affiliations:** Department of Cardiology, Beijing Anzhen Hospital, Capital Medical University, Institute of Heart Lung and Blood Vessel Disease, Beijing, 100029 China

**Keywords:** Triglyceride glucose index, Blood glucose, Glycosylated hemoglobin A, Acute coronary syndrome, Percutaneous coronary intervention, Prognosis

## Abstract

**Background:**

Previous studies have investigated the relationship of the triglyceride glucose (TyG) index with the incidence of cardiovascular events. However, to date, there have been no studies comparing the predictive values of fasting plasma glucose (FPG), glycosylated hemoglobin A (HbA1C) and the TyG index for the risk of cardiovascular events. This study aimed to use discordance analysis to evaluate and compare the effectiveness of FPG, HbA1C and the TyG index to predict the risk of cardiovascular events.

**Methods:**

Patients diagnosed with acute coronary disease (ACS) undergoing percutaneous coronary intervention (PCI) were enrolled in this study. The TyG index was computed using the following formula: ln [fasting triglycerides (mg/dL) × FPG (mg/dL)/2]. We categorized patients into 4 concordance/discordance groups. Discordance was defined as a TyG index equal to or greater than the median and an FPG or HbA1C less than the median, or vice versa. The primary outcome was the composite of death, nonfatal myocardial infarction, nonfatal stroke and unplanned repeat revascularization. A Cox proportional hazards regression model was performed to estimate the risk of cardiovascular events according to the concordance/discordance groups. Sensitivity analysis was performed on each patient group divided into high or low categories for HbA1C or FPG and were repeated according to diabetes status.

**Results:**

In total, 9285 patients were included in the final statistical analysis (male: 75.3%, age: 59.9 ± 10.05 years, BMI: 26.2 ± 9.21 kg/m^2^, diabetes: 43.9% and dyslipidemia: 76.8%). The medians defining concordance/discordance were 6.19 mmol/L for FPG, 6.1% for HbA1C and 8.92 for the TyG index. The TyG index was strongly related to triglycerides and HDL-C (r = 0.881 and -0.323, respectively; both P < 0.001). During the 17.4 ± 2.69 months of follow-up, there were 480 (5.1%) incident cardiovascular events. Among patients with a lower HbA1C or FPG, 15.6% and 16.3%, respectively, had a discordantly high TyG index and a greater risk of cardiovascular events compared with patients with a concordantly low TyG index after full adjustment (HR: 1.92, 95% CI 1.33–2.77; HR: 1.89, 95% CI 1.38–2.59; for HbA1C and FPG, respectively). Repeat risk estimation using high or low categories for FPG or HbA1C and diabetes status confirmed the results.

**Conclusions:**

Patients with a discordantly high TyG index had a significantly greater risk of cardiovascular events regardless of diabetes status. The TyG index might be a better predictor of cardiovascular risk than FPG or HbA1C for patients with ACS undergoing PCI. This discordance may support better cardiovascular risk management regardless of diabetes status.

## Background

Cardiovascular disease (CVD) is the leading cause of death worldwide [1, 2]. Although death related to CVD has decreased following the development of effective treatments, the rate of decline has slowed as a result of aging, obesity and diabetes mellitus (DM), urging us to examine risk factors more intensely [3]. Great progress has been made in understanding atherogenesis. The cholesterol hypothesis established an association between cholesterol and the risk of cardiovascular events. Decreasing low-density lipoprotein cholesterol (LDL-C) levels with statin [4], ezetimibe [5] and proprotein convertase subtilisin/kexin type 9(PCSK9) antibodies [6] resulted in an approximately 30–50% reduction of risk, though that still leaves a large degree of residual risk untreated [4]. Recently, inflammation inhibition has drawn much attention from cardiologists. For the first time, CANTOS (Canakinumab Anti-inflammatory Thrombosis Outcomes Study) showed that inhibition of the interleukin (IL)-1b pathway in patients with CAD could reduce the risk of cardiovascular events by approximately 17% [7]. Actually, insulin resistance (IR), which has a strong relationship with dyslipidemia and inflammation [8], has not received much attention. The residual risk after lipid-lowering agents could be attributed to IR to a large degree [9–11].

To our knowledge, there is no accepted convenient and efficient method for the diagnosis of IR. A hyperinsulinemic-euglycemic clamp is the gold standard diagnostic approach for IR, but it is difficult to use widely due to its cost and complexity [12]. Previous studies have introduced a variety of more convenient assessment methods for IR. The homeostatic model assessment of insulin resistance (HOMA-IR) is a relatively extensive method used in research [13], but the lack of standardized insulin assays has hindered its development [14]. The fasting triglyceride glucose (TyG) index, which includes fasting plasma glucose (FPG) and triglycerides (TG), has been proven to be significantly correlated with HOMA-IR and the hyperinsulinemic-euglycemic clamp (HIEC) [15, 16].

Many previous studies have investigated the relationship of the TyG index with the incidence of CVD and cardiovascular events in different patient groups, including both nondiabetic and diabetic patients [17–19]. Moreover, the TyG index has also been proven to be a better predictive factor than hemoglobin A1c for cardiovascular (CV) events in patients with Type 2 Diabetes Mellitus (T2DM) [20]. However, there are no studies comparing the capacity of the TyG index with FPG or glycosylated hemoglobin (HbA1C) to predict the risk of CV events in patients with acute coronary syndrome (ACS) undergoing percutaneous coronary intervention (PCI) regardless of diabetes status. Accordingly, this study aimed to use discordance analysis to evaluate and compare the effects of FPG, HbA1C and the TyG index to predict the risk of CV events.

## Methods

### Study design and patients

This retrospective cohort study consecutively enrolled 11916 patients if they were hospitalized for ACS and PCI from January 1, 2018, to January 31, 2019 at one of the top-ranked cardiovascular hospitals in China. Major exclusion criteria were a BMI > 45 kg/m^2^, severe hepatic and renal insufficiency (eGFR < 30 ml/min), heart failure (LVEF < 30%), cardiogenic shock, suspected familial hypertriglyceridemia (TG ≥ 5.65 mmol/L), fibrate use, pregnant and malignancy. The study protocol was approved by the institutional review board of Beijing Anzhen Hospital, Capital Medical University with a waiver of informed consent. Information related to the identities of the patients were concealed.

### Measurements

Data including patient demographics such as age, gender, BMI, smoking status, past medical history, laboratory results, PCI procedures, and medical treatments were obtained from hospital records. Blood samples were taken after overnight fasting (> 8 h). Serum levels of fasting plasma glucose (FPG), glycosylated hemoglobin (HbA1c) and lipid profiles, including TG, total cholesterol (TC), and high-density lipoprotein cholesterol (HDL-C), were determined by standard laboratory techniques. The enzymatic hexokinase method was used to measure FPG. TG was determined enzymatically and corrected for endogenous glycerol. The TyG index was computed using the following formula: ln [fasting TG (mg/dL) × FPG (mg/dL)/2] [15]. The low-density lipoprotein cholesterol (LDL-C) level was computed with the Friedewald equation. For patients with diabetes, two or more FPG measurements were taken and the mean value was used for the final analysis.

### Treatment and Procedure

The PCI operation and medication were implemented according to relevant guidelines [21]. All patients were given aspirin and clopidogrel or ticagrelor before operation and 70–100 IU/kg unfractionated heparin during the operation whenever appropriate. PCI was performed using 6 or 7 Fr guiding catheters via a radial approach. Predilatation and second-generation drug eluting stents were preferred whenever possible. The use of FFR, IVUS, OCT and the type of stent were at the discretion of the clinicians.

### Outcomes

Relevant information regarding cardiovascular events was also collected from hospital records for readmitted patients. All patients were followed up for at least 12 months by telephone interviews, and only index events were included in the statistical analysis for repeated events. Clinical events were recorded doubly and inconsistent events were affirmed by a third record. The primary outcome was the composite of death, nonfatal MI, nonfatal stroke or unplanned repeat revascularization. The definition of myocardial infarction is derived from the fourth universal definition of that term [22]. Death was defined as all causes of death regardless of the cause of death [23]. Stroke was adjudicated by the presence of acute infarction as demonstrated by the persistence of symptoms or imaging [23]. Unplanned repeat revascularization, including target lesion revascularization and target vessel revascularization, were defined as any repeat percutaneous intervention or bypass surgery of the target lesions or vessels [23, 24]. Unstable angina is defined as myocardial ischemia at rest or minimal exertion in the absence of cardiomyocyte necrosis [25]. Acute myocardial infarction (MI) is defined as the presence of acute myocardial injury detected by abnormal cardiac biomarkers in the setting of evidence of acute myocardial ischemia [22]. Hypertension was defined as a systolic BP ≥ 140 mm Hg, diastolic BP ≥ 90 mm Hg, or the use of antihypertensive medications [26]. Diabetes mellitus was defined as a fasting (≥ 8 h) serum glucose ≥ 7.0 mmol/L, a nonfasting glucose ≥ 11.10 mmol/L and the use of hypoglycemic agents [27]. Dyslipidemia was defined as a fasting TC > 5.18 mmol/L(200 mg/dL), LDL-C > 3.37 mmol/L(130 mg/dL), TG > 1.72 mmol/L(150 mg/dL), HDL-C<1.0 mmol/L(40 mg/dL), or the use of lipid-lowering drugs.

### Statistical analyses

All statistical analyses were conducted with the SPPS 24.0 software (IBM Corp., Armonk, NY, USA). A two-tailed p value < 0.05 was required for statistical significance. First, the correlation between the TyG index and lipid parameters was computed using the Pearson or Spearman rank correlation. We then calculated the medians for the FPG, HbA1C and TyG index to classify patients into the following 2 categories: low (less than the medians) and high (equal to or greater than the medians). Finally, we categorized patients into 4 groups according to having a low or high TyG index and FPG or HbA1C, as follows: low/low, low/high, high/low, and high/high. Discordance was defined as a high TyG index and the FPG or HbA1C being low, or vice versa. We preferred to use the median as the cut-off point value over the guideline-guide target value because all patients without diabetes would fall into the low group, and we would be unable to take full advantage of all the information.

Baseline characteristics were compared according to the 4 concordance/discordance groups. Continuous variables were presented as the mean ± standard deviation (SD) or median (interquartile range) with significance tests performed by one-way analysis of variance (ANOVA) or the Kruskal–Wallis H test. When applicable, post hoc analyses were performed using the Bonferroni method. Categorical variables were displayed as numbers (percentage) with significance tests performed using the Chi squared test or Fisher’s exact test. For cardiovascular event outcomes, the log-rank test and backward stepwise selection methods in a Cox proportional hazards regression model were performed with minimally and fully adjusted univariate and multivariable analyses. The following three models were used for the multivariate analysis: Model 1 (minimally adjusted): age, sex, and BMI; Model 2: model 1 + current smoker, hypertension, previous MI, previous stroke, previous PCI, previous CABG, and ACS status; and Model 3 (fully adjusted): Model 2 + non-HDL-C and lipid-lowering and anti-diabetes medication use. Additionally, we repeated the discordance analysis for each group of patients divided into the high or low categories for HbA1C or FPG. Finally, analyses of the risk of cardiovascular events across the 4 concordance/discordance groups were repeated according to diabetes status. However, different cut-off values that defined concordance/discordance were used when considering diabetes status. Median values were used for nondiabetic patients and guideline-guide target values for HbA1C and an equivalent FPG percentage and TyG index were used for patients with diabetes.

## Results

**Fig. 1:**
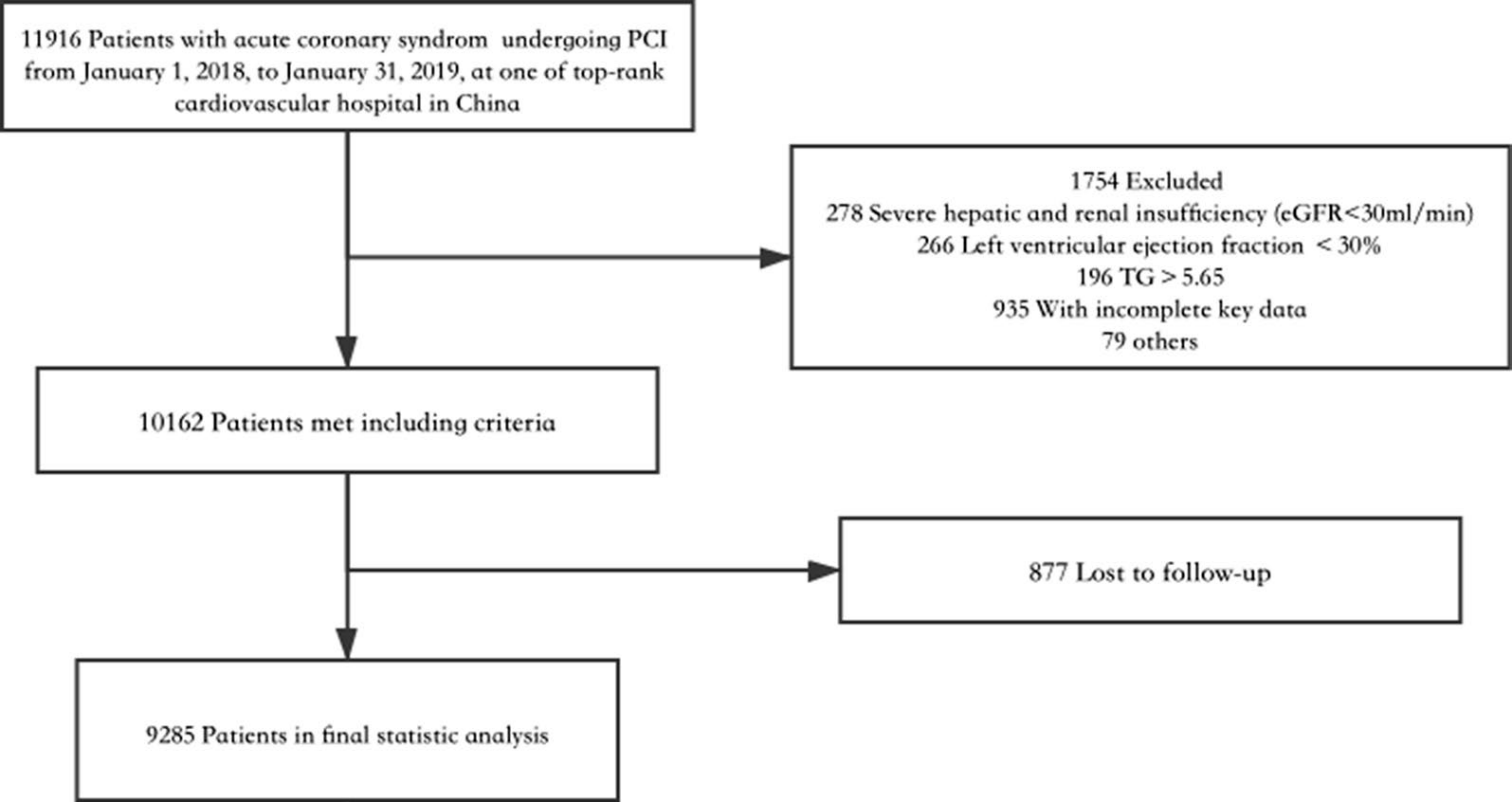
Flowchart

Among the 11916 consecutive patients with ACS who underwent PCI, 1754 patient who met the major exclusion criteria and 877 patients lost during follow-up were excluded. In total, 9285 patients were included in the final analysis. A flowchart for determining the patients for the final analysis is displayed in Fig. 1. When we compared the patients’ baseline characteristics, we found that there were almost no significant differences between the lost participants and eligible participants(Additional file 1: Table S1). For blood pressure and heart rate, there were small and statistically significant but not clinically relevant differences.

The baseline characteristics are shown in Tables 1 and 2. Among the included patients, 6996 (75.3%) were male with a mean age of 59.9 ± 10.05 years and BMI of 26.2 ± 9.21 kg/m^2^, 43.9% (4074) and 76.8% (7131) of whom had diabetes and dyslipidemia, respectively. Most of the participants presented unstable angina (84.9%), while a small subset of patients showed ST segment elevation myocardial infarction (STEMI) and non-ST segment elevation myocardial infarction (NSTEMI). The medians that defined concordance/discordance were 6.19 mmol/L for FPG, 6.1% for HbA1C and 8.92 for the TyG index. Antidiabetic agents including oral antidiabetic agents and insulin were given to 3314 (35.7%) patients who accounted for 81.3% of the patients with diabetes. Moreover, 98.2% of patients were prescribed statin and 18.4% patients with ezetimibe. At procedural baseline, angiographic coronary lesions were complex, with 15.7% left main lesions and 58.3% multivessel lesions. FFR, IVUS and OCT were not widely used on the participants.

**Table 1:** Characteristics of the concordance/discordance groups according to low or high HbA1C and TyG index categories

HbA1C/TyG^a^	Total	Low/Low	Low/High	High/Low	High/High	*P value*^*†*^
Concordance/discordance groups						
N (%)	9285(100)	2713(29.2)	1516(16.3)	1925(20.7)	3131(33.7)	–
Age, year	59.9 ± 10.05	59.3 ± 10.35	56.7 ± 10.35	63.2 ± 8.89	59.9 ± 9.72	< 0.001
Male, n (%)	6996 (75.3)	2176 (80.2)	1177 (77.6)	1437 (74.6)	2206 (70.5)	< 0.001
BMI, kg/m2	26.2 ± 9.21	25.5 ± 9.91	26.6 ± 6.58	26.0 ± 8.46	26.9 ± 10.04	< 0.001
Heart rate, bpm	72 ± 12.72	70.4 ± 12.94	72.3 ± 12.98	71.6 ± 11.72	73.5 ± 12.81	< 0.001
SBP, mmHg	128.6 ± 20.86	128.2 ± 19.87	127.2 ± 21.12	128.7 ± 21.68	129.5 ± 21.03	0.004
Medical history and risk factors, n (%)						
Current smoker	3497 (37.7)	1055 (38.9)	624 (41.2)	641 (33.3)	1177 (37.6)	< 0.001
Hypertension	6104 (65.7)	1608 (59.3)	937 (61.8)	1325 (68.8)	2234 (71.4)	< 0.001
Diabetes	4074 (43.9)	136 (5)	106 (7)	1213 (63)	2619 (83.6)	< 0.001
Dyslipidaemia	7131 (76.8)	2046 (75.4)	1206 (79.6)	1413 (73.4)	2466 (78.8)	< 0.001
Previous MI	1156 (12.5)	292 (10.8)	165 (10.9)	251 (13)	448 (14.3)	< 0.001
Previous stroke	485 (5.2)	139 (5.1)	67 (4.4)	110 (5.7)	169 (5.4)	0.367
Previous PCI	2264 (24.4)	545 (20.1)	304 (20.1)	526 (27.3)	889 (28.4)	< 0.001
Previous CABG	220 (2.4)	42 (1.5)	27 (1.8)	58 (3)	93 (3)	< 0.001
Laboratory tests						
FPG, mmol/L	7.1 ± 2.61	5.5 ± 0.8	6.1 ± 1.56	6.6 ± 1.55	9.1 ± 3.14	< 0.001
HbA1C, %	6.6 ± 1.39	5.6 ± 0.31	5.6 ± 0.31	7 ± 1.04	7.8 ± 1.46	< 0.001
TC, mmol/L	4.1 ± 1.07	4 ± 1	4.5 ± 1.07	3.8 ± 0.95	4.3 ± 1.1	< 0.001
TG, mmol/L	1.43(1.03-2.07)	1.1(0.86-1.35)	2.2(1.87-2.75)	1(0.79-1.22)	1.96(1.52-2.68)	< 0.001
HDL-C, mmol/L	1.1 ± 0.25	1.1 ± 0.27	1 ± 0.23	1.1 ± 0.26	1 ± 0.22	< 0.001
LDL-C, mmol/L	2.4 ± 0.88	2.4 ± 0.88	2.6 ± 0.92	2.2 ± 0.82	2.5 ± 0.88	< 0.001
Non-HDL-C, mmol/L	3.1 ± 1.02	2.8 ± 0.93	3.5 ± 1.01	2.7 ± 0.87	3.3 ± 1.05	< 0.001
TyG index	9 ± 0.67	8.4 ± 0.35	9.3 ± 0.37	8.5 ± 0.31	9.6 ± 0.54	< 0.001
ACS status, n (%)						
Unstable angina	7883 (84.9)	2293 (84.5)	1231 (81.2)	1718 (89.2)	2641 (84.4)	< 0.001
NSTEMI	1152 (12.4)	330 (12.2)	224 (14.8)	178 (9.2)	420 (13.4)	< 0.001
STEMI	829 (8.9)	260 (9.6)	173 (11.4)	130 (6.8)	266 (8.5)	< 0.001
Medication at discharge, n (%)						
Metformin	807 (8.7)	21 (0.8)	16 (1.1)	187 (9.7)	583 (18.6)	< 0.001
Sulfonylurea	469 (5.1)	16 (0.6)	8 (0.5)	131 (6.8)	314 (10)	< 0.001
Alpha-glucosidase inhibitors	1656 (17.8)	52 (1.9)	36 (2.4)	464 (24.1)	1104 (35.3)	< 0.001
Thiazolidinedione	20 (0.2)	3 (0.1)	1 (0.1)	6 (0.3)	10 (0.3)	0.147
Dipeptidyl peptidase 4 inhibitors	53 (0.6)	4 (0.1)	1 (0.1)	16 (0.8)	32 (1)	< 0.001
Oral antidiabetic agents	2108 (22.7)	67 (2.5)	47 (3.1)	578 (30)	1416 (45.2)	< 0.001
Insulin	1728 (18.6)	14 (0.5)	24 (1.6)	475 (24.7)	1215 (38.8)	< 0.001
Any antidiabetic agents	3314 (35.7)	75 (2.8)	65 (4.3)	920 (47.8)	2254 (72)	< 0.001
Aspirin	9062 (97.6)	2656 (97.9)	1484 (97.9)	1879 (97.6)	3043 (97.2)	0.281
Clopidogrel	6316 (68)	1888 (69.6)	969 (63.9)	1368 (71.1)	2091 (66.8)	< 0.001
Ticagrelor	3131 (33.7)	883 (32.5)	562 (37.1)	596 (31)	1090 (34.8)	0.001
ACEI/ARB	4165 (44.9)	1042 (38.4)	688 (45.4)	871 (45.2)	1564 (50)	< 0.001
β-Blocker	5980 (64.4)	1619 (59.7)	997 (65.8)	1204 (62.5)	2160 (69)	< 0.001
Statin	9114 (98.2)	2671 (98.5)	1482 (97.8)	1897 (98.5)	3064 (97.9)	0.123
Ezetimibe	1712 (18.4)	460 (17)	327 (21.6)	304 (15.8)	621 (19.8)	< 0.001
Angiographic coronary anatomy, n (%)						
Any left main disease	1459 (15.7)	426 (15.7)	235 (15.5)	316 (16.4)	482 (15.4)	0.799
Multivessel disease	5412 (58.3)	1157 (42.6)	903 (59.6)	1147 (59.6)	2205 (70.4)	< 0.001
Others	3564 (38.4)	1448 (53.4)	540 (35.6)	720 (37.4)	856 (27.3)	< 0.001
CTO	1499 (16.1)	367 (13.5)	277 (18.3)	314 (16.3)	541 (17.3)	< 0.001
Lesions > 20 mm	5640 (60.7)	1111 (41)	853 (56.3)	1187 (61.7)	2489 (79.5)	< 0.001
Treated vessel, n (%)						
LM	894 (9.6)	276 (10.2)	134 (8.8)	198 (10.3)	286 (9.1)	0.279
LAD	4735 (51)	1426 (52.6)	728 (48)	1017 (52.8)	1564 (50)	0.007
LCX	2693 (29)	774 (28.5)	471 (31.1)	520 (27)	928 (29.6)	0.051
RCA	3704 (39.9)	1014 (37.4)	625 (41.2)	785 (40.8)	1280 (40.9)	0.017
DCB	567 (6.1)	164 (6)	72 (4.7)	131 (6.8)	200 (6.4)	0.073
FFR	67 (0.7)	26 (1)	8 (0.5)	19 (1)	14 (0.4)	0.044
IVUS	247 (2.7)	65 (2.4)	48 (3.2)	53 (2.8)	81 (2.6)	0.501
OCT	156 (1.7)	59 (2.2)	28 (1.8)	23 (1.2)	46 (1.5)	0.049
Number of stents	2 (1–3)	2 (1–3)	2 (1–3)	2 (1–3)	2 (1–3)	0.075
Total length of stents, mm	32 (21–52)	30 (21–51)	32 (20.5–52)	33 (21–51)	33 (21–54)	0.417

Values are mean ± SD, median (interquartile range), or n (%)

*BMI* body mass index, *SBP* systolic blood pressure, *MI* myocardial infarction, *PCI* percutaneous coronary intervention, *CABG* Coronary Artery Bypass Grafting, *FPG* fasting plasma glucose, *HbA1C* Glycosylated haemoglobin, *TC* total cholesterol, *HDL-C* high-density lipoprotein-cholesterol, *LDL-C* low-density lipoprotein-cholesterol, *TyG* triglyceride glucose, *NSTEMI* non ST-segment elevation myocardial infarction, *STEMI ST*-segment elevation myocardial infarction, *ACEI* angiotensin converting enzyme inhibitor, *ARB* angiotensin II receptor blocker, *CTO* chronic total occlusion, *LM* left-main artery, *LAD* left anterior descending artery, *LCX* left circumflex artery, *RCA* right coronary artery, *DCB* drug-coated balloon, *FFR* Fractional Flow Reserve, *IVUS* intravascular ultrasound, *OCT* optical coherence tomography

^a^Median HbA1C: 6.1%, Median TyG:8.92

†p value for test of difference across the 4 concordance/discordance groups by the Chi square test for categorical variables or analysis of variance for continuous variables or Kruskal–Wallis test for nonparametric comparisons

**Table 2:** Characteristics of the concordance/discordance groups according to low or high FPG and TyG index categories

FBG/TyG^a^	Total	Low/Low	Low/High	High/Low	High/High	*P* value^†^
Concordance/discordance groups						
N (%)	9285 (100)	3194 (34.4)	1446 (15.6)	1444 (15.6)	3201 (34.5)	–
Age, year	59.9 ± 10.05	60.1 ± 10.15	57 ± 10.57	62.6 ± 9.28	59.7 ± 9.68	< 0.001
Male, n (%)	6996 (75.3)	2505 (78.4)	1083 (74.9)	1108 (76.7)	2300(71.9)	< 0.001
BMI, kg/m2	26.2 ± 9.21	25.7 ± 9.24	26.7 ± 3.25	25.8 ± 9.57	26.8 ± 10.72	< 0.001
Heart rate, bpm	72 ± 12.72	70.6 ± 11.35	72 ± 12.05	71.3 ± 14.61	73.6 ± 13.2	< 0.001
SBP, mmHg	128.6 ± 20.86	128.1 ± 20.39	127.8 ± 21.92	129 ± 21.19	129.1 ± 20.68	0.1
Medical history and risk factors, n (%)						
Current smoker	3497 (37.7)	1211 (37.9)	581 (40.2)	485 (33.6)	1220 (38.1)	0.002
Hypertension	6104 (65.7)	1957 (61.3)	943 (65.2)	976 (67.6)	2228 (69.6)	< 0.001
Diabetes	4074 (43.9)	444 (13.9)	230 (15.9)	905 (62.7)	2495 (77.9)	< 0.001
Dyslipidaemia	7131 (76.8)	2386 (74.7)	1143 (79)	1073 (74.3)	2529 (79)	< 0.001
Previous MI	1156 (12.5)	364 (11.4)	148 (10.2)	179 (12.4)	465 (14.5)	< 0.001
Previous stroke	485 (5.2)	153 (4.8)	55 (3.8)	96 (6.6)	181 (5.7)	0.003
Previous PCI	2264 (24.4)	722 (22.6)	307 (21.2)	349 (24.2)	886 (27.7)	< 0.001
Previous CABG	220 (2.4)	61 (1.9)	35 (2.4)	39 (2.7)	85 (2.7)	0.191
Laboratory tests						
FPG, mmol/L	7.1 ± 2.61	5.3 ± 0.49	5.5 ± 0.45	7.4 ± 1.29	9.4 ± 2.99	< 0.001
HbA1C, %	6.6 ± 1.39	5.9 ± 0.68	5.9 ± 0.63	6.8 ± 1.22	7.6 ± 1.59	< 0.001
TC, mmol/L	4.1 ± 1.07	4 ± 1	4.5 ± 1.09	3.8 ± 0.92	4.3 ± 1.09	< 0.001
TG, mmol/L	1.43 (1.03–2.07)	1.12 (0.86–1.38)	2.29 (1.96–2.83)	0.95 (0.76–1.1275)	1.9 (1.49–2.62)	< 0.001
HDL-C, mmol/L	1.1 ± 0.25	1.1 ± 0.27	1 ± 0.22	1.1 ± 0.26	1 ± 0.22	< 0.001
LDL-C, mmol/L	2.4 ± 0.88	2.4 ± 0.89	2.6 ± 0.94	2.2 ± 0.79	2.5 ± 0.87	< 0.001
Non-HDL-C, mmol/L	3.1 ± 1.02	2.8 ± 0.93	3.5 ± 1.02	2.6 ± 0.82	3.3 ± 1.04	< 0.001
TyG index	9 ± 0.67	8.4 ± 0.35	9.3 ± 0.33	8.6 ± 0.27	9.6 ± 0.54	< 0.001
ACS status, n (%)						
Unstable angina	7883 (84.9)	2795 (87.5)	1214 (84)	1216 (84.2)	2658 (83)	< 0.001
NSTEMI	1152 (12.4)	326 (10.2)	191 (13.2)	182 (12.6)	453 (14.2)	< 0.001
STEMI	829 (8.9)	250 (7.8)	129 (8.9)	140 (9.7)	310 (9.7)	0.045
Medication at discharge, n (%)						
Metformin	807 (8.7)	56 (1.8)	33 (2.3)	152 (10.5)	566 (17.7)	< 0.001
Sulfonylurea	469 (5.1)	34 (1.1)	17 (1.2)	113 (7.8)	305 (9.5)	< 0.001
Alpha-glucosidase inhibitors	1656 (17.8)	140 (4.4)	54 (3.7)	376 (26)	1086 (33.9)	< 0.001
Thiazolidinedione	20 (0.2)	7 (0.2)	0 (0)	2 (0.1)	11 (0.3)	0.113
Dipeptidyl peptidase 4 inhibitors	53 (0.6)	5 (0.2)	2 (0.1)	15 (1)	31 (1)	< 0.001
Oral antidiabetic agents	2108 (22.7)	179 (5.6)	70 (4.8)	466 (32.3)	1393 (43.5)	< 0.001
Insulin	1728 (18.6)	146 (4.6)	50 (3.5)	343 (23.8)	1189 (37.1)	< 0.001
Any antidiabetic agents	3314 (35.7)	282 (8.8)	113 (7.8)	713 (49.4)	2206 (68.9)	< 0.001
Aspirin	9062 (97.6)	3127 (97.9)	1414 (97.8)	1408 (97.5)	3113 (97.3)	0.365
Clopidogrel	6316 (68)	2260 (70.8)	969 (67)	996 (69)	2091 (65.3)	< 0.001
Ticagrelor	3131 (33.7)	1002 (31.4)	497 (34.4)	477 (33)	1155 (36.1)	< 0.001
ACEI/ARB	4165 (44.9)	1241 (38.9)	650 (45)	672 (46.5)	1602 (50)	< 0.001
β-Blocker	5980 (64.4)	1904 (59.6)	923 (63.8)	919 (63.6)	2234 (69.8)	< 0.001
Statin	9114 (98.2)	3147 (98.5)	1408 (97.4)	1421 (98.4)	3138 (98)	0.043
Ezetimibe	1712 (18.4)	498 (15.6)	274 (18.9)	266 (18.4)	674 (21.1)	< 0.001
Angiographic coronary anatomy, n (%)						
Any left main disease	1459 (15.7)	527 (16.5)	226 (15.6)	215 (14.9)	491 (15.3)	0.461
Multivessel disease	5412 (58.3)	1429 (44.7)	847 (58.6)	875 (60.6)	2261 (70.6)	< 0.001
Others	3564 (38.4)	1630 (51)	541 (37.4)	538 (37.3)	855 (26.7)	< 0.001
CTO	1499 (16.1)	441 (13.8)	251 (17.4)	240 (16.6)	567 (17.7)	< 0.001
Lesions > 20 mm	5640 (60.7)	1434 (44.9)	842 (58.2)	864 (59.8)	2500 (78.1)	< 0.001
Treated vessel, n (%)						
LM	894 (9.6)	345 (10.8)	130 (9)	129 (8.9)	290 (9.1)	0.052
LAD	4735 (51)	1686 (52.8)	680 (47)	757 (52.4)	1612 (50.4)	0.002
LCX	2693 (29)	910 (28.5)	456 (31.5)	384 (26.6)	943 (29.5)	0.025
RCA	3704 (39.9)	1207 (37.8)	584 (40.4)	592 (41)	1321 (41.3)	0.026
DCB	567 (6.1)	214 (6.7)	79 (5.5)	81 (5.6)	193 (6)	0.3
FFR	67 (0.7)	35 (1.1)	9 (0.6)	10 (0.7)	13 (0.4)	0.012
IVUS	247 (2.7)	77 (2.4)	46 (3.2)	41 (2.8)	83 (2.6)	0.472
OCT	156 (1.7)	59 (1.8)	30 (2.1)	23 (1.6)	44 (1.4)	0.286
Number of stents	2 (1–3)	2 (1–3)	2 (1–3)	2 (1–3)	2 (1–3)	0.044
Total length of stents, mm	32 (21–52)	30 (21–51)	33 (22–54)	30 (20–51)	32 (21–53)	0.219

Values are mean ± SD, median (interquartile range), or n (%)

*BMI* body mass index, *SBP* systolic blood pressure, *MI* myocardial infarction, *PCI* percutaneous coronary intervention, *CABG* Coronary Artery Bypass Grafting, *FPG* fasting plasma glucose, *HbA1C* Glycosylated haemoglobin, *TC* total cholesterol, *HDL-C* high-density lipoprotein-cholesterol, *LDL-C* low-density lipoprotein-cholesterol, *TyG* triglyceride FBGcose, *NSTEMI* non ST-segment elevation myocardial infarction, *STEMI* ST-segment elevation myocardial infarction, *ACEI* angiotensin converting enzyme inhibitor, *ARB* angiotensin II receptor blocker, *CTO* chronic total occlusion, *LM* left-main artery, *LAD* left anterior descending artery, *LCX* left circumflex artery, *RCA* right coronary artery, *DCB* drug-coated balloon, *FFR* Fractional Flow Reserve, *IVUS* intravascular ultrasound, *OCT* optical coherence tomography

^†^p value for test of difference across the 4 concordance/discordance groups by the Chi square test for categorical variables or analysis of variance for continuous variables or Kruskal–Wallis test for nonparametric comparisons

^a^Median FBG: 6.19 mmol/L, Median TyG:8.92

The baseline characteristics were compared across the 4 concordance/discordance groups according to low or high categories for HbA1C and the TyG index, as shown in Table 1. Among those with a lower HbA1C or FPG, 16.3% and 15.6% patients had a discordantly high TyG index, respectively. There were significant differences in age, gender, current smoker, diabetes, dyslipidemia and antidiabetic treatment across the 4 groups. There were small and statistically significant but not clinically relevant differences in heart rate and SBP. Furthermore, patients with a high TyG index were more likely to have a higher TG, LDL-C, non-HDL-C and lower HDL-C compared with the low TyG index group and had more multivessel and longer coronary lesions. Similar patterns were also observed in the FPG/TyG index analyses shown in Table 2.

A correlation analysis showed that the TyG index is strongly related to TG and HDL-C (r = 0.881 and −0.323, respectively; both P<0.001). During the 17.4 ± 2.69 months of follow-up, there were 480 incident cardiovascular events, accounting for 5.1% of all patients. Table 3 shows the hazard risks of cardiovascular events in patients using tertiles for the FPG, HbA1C, TyG index and 4 concordance/discordance groups. Compared with the low tertile, the middle (hazard ratio (HR): 1.72; 95% confidence interval (CI) 1.29−2.28) and high (HR: 2.70; 95% CI 2.05–3.54) HbA1C tertile was significantly increased risk after adjustment with model 3. Using the lowest tertile for the TyG index as a reference, patients in the middle and high tertiles had a greater risk of cardiovascular events in the multivariable-adjusted model (HR: 1.33, 95% CI 1.01–1.76; HR: 2.36, 95% CI 1.82–3.07). For the FPG tertiles, the middle tertile did not show a significantly greater risk compared with the low tertile.

**Table 3:** Risk of cardiovascular events using the FPG, HbA1C, TyG index and concordance/discordance groups

	HR (95% CI)			
	Unadjusted	Model 1^a^	Model 2^a^	Model 3^a^
FPG tertiles				
Q1 (referent)	–	–	–	–
Q2	0.99 (0.78–1.27)	1.00 (0.78–1.29)	1.00 (0.77–1.30)	0.98 (0.75–1.26)
Q3	1.82 (1.46-2.26)^†^	1.81 (1.44-2.26)^†^	1.80 (1.43-2.27)^†^	1.76 (1.39-2.21)^†^
HbA1C tertiles				
Q1 (referent)	–	–	–	–
Q2	1.75 (1.31–3.79)^†^	1.78 (1.34–2.36)^†^	1.79 (1.34–2.39)^†^	1.72 (1.29–2.28)^†^
Q3	2.92 (2.24–3.79)^†^	2.85 (2.18–3.73)^†^	2.89 (2.20–3.80)^†^	2.70 (2.05–3.54)^†^
TyG tertiles				
Q1 (referent)	–	–	–	–
Q2	1.42 (1.09–1.86)^‡^	1.46 (1.11–1.92)^‡^	1.46 (1.01–1.92)^§^	1.33 (1.01–1.76)^§^
Q3	2.80 (2.21–3.55)^†^	2.81 (2.20–3.60)^†^	2.85 (2.22–3.67)^†^	2.36 (1.82–3.07)^†^
FPG/TyG^a^				
Low/low (referent)	–	–	–	–
Low/High	2.10 (1.56–2.83)^†^	2.21 (1.63–2.99)^†^	2.22 (1.63–3.03)^†^	1.89 (1.38–2.59)^†^
High/Low	1.41 (1.01–1.96)^§^	1.45 (1.03–2.04)^§^	1.43 (1.01–2.03)^§^	1.52 (1.07–2.15)^§^
High/High	2.83 (2.22–3.60)^†^	2.81 (2.19–3.60)	2.83 (2.20–3.65)^†^	2.48 (1.92–3.21)^†^
HbA1C/TyG^a^				
Low/low (referent)	–	–	–	–
Low/High	2.13 (1.50–3.01)^†^	2.20 (1.54–3.13)^†^	2.18 (1.52–3.12)^†^	1.92 (1.33–2.77)^†^
High/Low	2.12 (1.52–2.95)^†^	2.12 (1.51–2.97)^†^	2.11 (1.50–2.97)^†^	2.28 (1.61–3.23)^†^
High/High	3.99 (3.01–5.29)^†^	3.93 (2.94–5.26)^†^	3.99 (2.98–5.36)^†^	3.79 (2.78–5.17)^†^

*FPG* fasting plasma glucose, *HbA1C* Glycosylated haemoglobin, *TyG* triglyceride glucose, *HR* hazard ratio, *CI* confidence interval

^†^P<0.001

^‡^P<0.01

^§^P<0.05

^a^The log-rank test and backward stepwise selection methods in a Cox proportional hazards regression model was performed; Model 1: adjusted for age, sex, BMI; Model 2: Model 1 + current smoker, hypertension, previous MI, previous stroke, previous PCI, previous CABG, ACS status; Model 3: Model 2 + non-HDL-C, lipid-lowering and antidiabetes medication use. Median HbA1C: 6.1%, median FBG: 6.19 mmol/L, median TyG:8.92

**Fig. 2:**
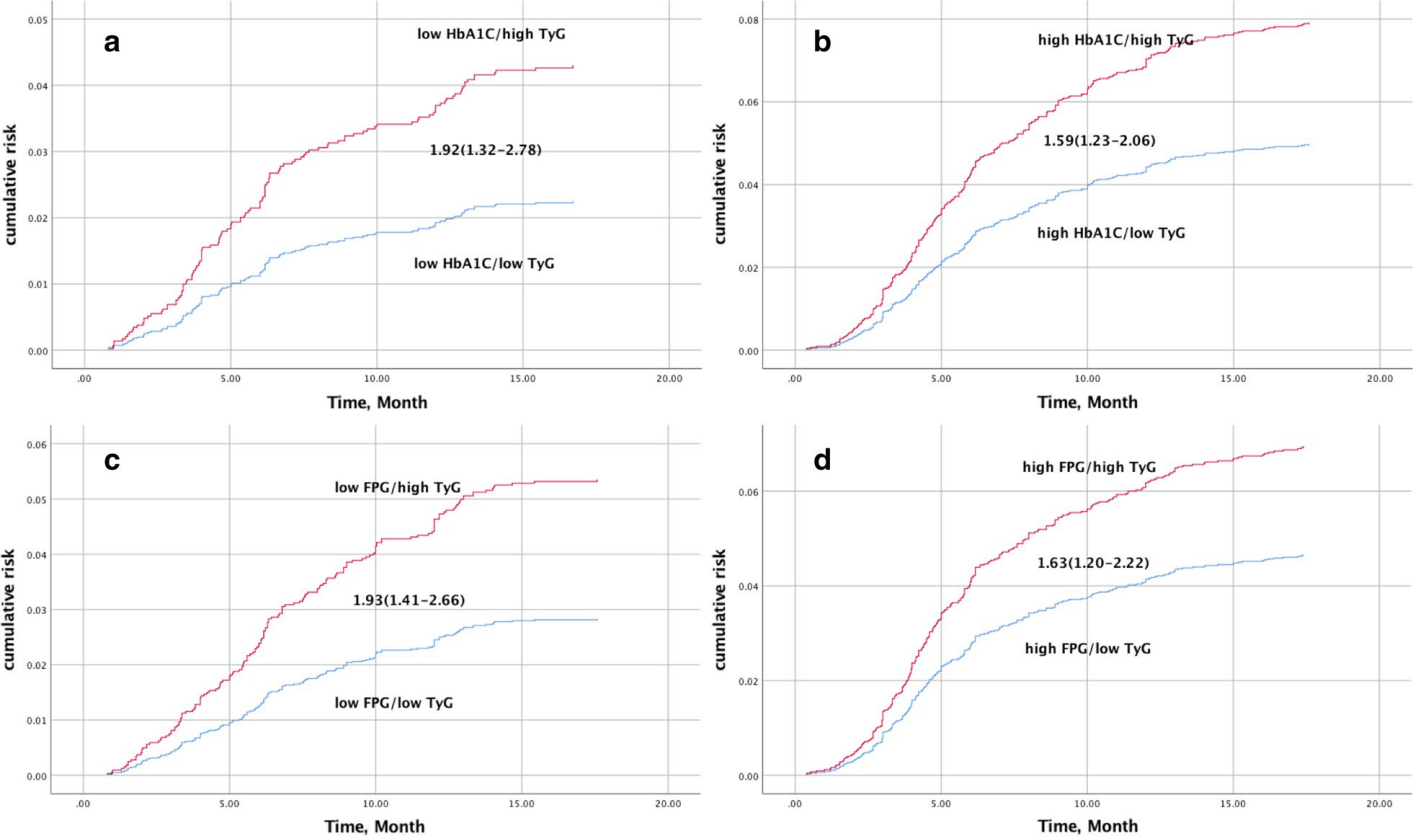
Concordance/discordance analysis of the risk of cardiovascular events in patients with low HbA1C (**a**), high HbA1C (**b**), low FPG (**c**), and high FPG (**d**)

Considering the discordance analysis (Table 3), among those with lower HbA1C or FPG, patients with a discordantly high TyG index had a greater risk of cardiovascular events compared to those with a concordantly low TyG index after fully adjustment (HR: 1.92, 95% CI 1.33–2.77; HR: 1.89, 95% CI 1.38–2.59; for HbA1C and FPG, respectively). Similarly, among patients with a high HbA1C or FPG, those with a discordantly low TyG index had a lower risk of cardiovascular events compared with those with a concordantly high TyG index. The group with a high TyG index and concordantly high FPG or HbA1C had the highest risk (HR: 2.48, 95% CI 1.92–3.21; HR: 3.79, 95% CI 2.78–5.17; for FPG and HbA1C, respectively). The pattern of results was similar for the repeated discordance analysis when each patient group was divided into high or low categories for HbA1C or FPG (Fig. 2).

Table 4 calculates the risk of cardiovascular events in the concordance/discordance groups by diabetes status. Different cut-off values for concordance/discordance were used when accounting for diabetes status. In the non-DM group, a median HbA1C of 5.8%, median FPG of 5.52 mmol/L, and median TyG index of 8.74 were used. In the DM group, the guideline-guide HbA1C target of 7.0%, an equivalent FPG percentage of 7.11 mmol/L and an equivalent TyG index percentage of 8.95 were used. This revealed that patients with a discordantly high TyG index had a significantly greater risk of cardiovascular events regardless of their diabetes status.

**Table 4:** Risk of cardiovascular events across concordance/discordance groups stratified by diabetes status

Non-DM^a^			DM^a^	
HbA1C/TyG^†^	HR (95%CI)	*P value*	HR (95%CI)	*P value*
Low/low (referent)	–	< 0.001	–	–
Low/High	1.91 (1.14–3.20)	0.014	2.10 (1.41–3.12)	< 0.001
High/Low	1.54 (0.90–2.62)	0.116	1.88 (1.26–2.81)	0.002
High/High	3.22 (2.02–5.16)	< 0.001	2.79(1.98–3.93)	< 0.001
FPG/TyG^†^	HR (95%CI)	*P value*	HR (95%CI)	*P value*
Low/low (referent)	–	< 0.001	–	–
Low/High	1.73 (1.23–2.45)	0.002	1.62 (1.01–2.62)	0.047
High/Low	0.40 (0.06–2.93)	0.369	1.4 3 (0.87–2.34)	0.161
High/High	3.56 (1.65–7.66)	0.001	1.99 (1.36–2.91)	< 0.001

*FPG* fasting plasma glucose, *HbA1C* Glycosylated haemoglobin, *TyG* triglyceride glucose, *HR* hazard ratio, *CI* confidence interval

^†^Different cutoff values were used by diabetes status. In non-DM group: Median HbA1C: 5.8%, median FBG: 5.52 mmol/L, median TyG: 8.74; In DM Group: guideling-guide HbA1C: 7.0%, equivalent percentage FPG: 7.11 mmol/L, equivalent percentage TyG:8.95

^a^The log-rank test and backward stepwise selection methods in a Cox proportional hazards regression model was performed; adjusted for age, sex, BMI, current smoker, hypertension, previous MI, previous stroke, previous PCI, previous CABG, non-HDL-C, lipid-lowering and antidiabetes medication use

## Discussion

In Chinese patients with ACS undergoing PCI, we observed that discordance defined as the median between the TyG index and FPG or HbA1C was common regardless of diabetes status, in approximately 15% of people. Patients with a high TyG index were more likely to be have a high TG, LDL-C, non-HDL-C and low HDL-C, which is strongly related to TG and HDL-C. In addition, we also demonstrated that patients with a discordantly high TyG index had a significantly greater risk of cardiovascular events regardless of their diabetes status and independent of traditional CVD risk factors, even after adjusting for non-HDL-C. Specifically, the risk of cardiovascular events may be either overestimated or underestimated when using HbA1C or FPG alone. To our knowledge, our study is the first comparing the effectiveness of the FPG, HbA1C and TyG index to predict the risk of cardiovascular events in patients with ACS undergoing PCI.

These data support that the TyG index, a factor easily available from a common lipid profile at no extra cost, is a better predictive factor of cardiovascular risks than FPG or HbA1C and provides additional information with important clinical significance. In patients without diabetes, the TyG index can identify patients with a higher risk of cardiovascular events, which may promote more positive therapies such as exercise, diet management, and even medication treatment to reduce risk. For patients with diabetes, even if patients have reached the guideline-guide targets for HbA1C and FPG, the TyG index can identify patients with an increased risk and prompt patients to consider more positive treatments to further reduce their risk. More importantly, the TyG index can be used as an additional target other than HbA1C and FPG to further reduce risk and promote the development of more drugs to improve IR.

Several studies have demonstrated that the TyG index is closely correlated with cardiovascular risk in different patients. Patients with a higher TyG index were more likely to develop hypertension [28, 29], diabetes [30], and obstructive sleep apnea [31] than those with a lower TyG index. Moreover, recent studies have shown that the TyG index is associated with subclinical cerebral and coronary artery disease. In neurologically healthy patients, a higher TyG index was associated with a higher incidence of subclinical cerebral small vessel disease (cSVD) [32]. A study including healthy patients demonstrated that the TyG index was an independent marker for predicting subclinical CAD, which is defined as the presence of any coronary plaque on coronary computed tomographic angiography [33]. A recent study also showed that the TyG index was correlated with arterial stiffness measured by brachial–ankle pulse wave velocity (baPWV) in both men and women [34] and coronary artery calcium (CAC) progression [35]. Furthermore, in healthy participants, patients with a high TyG index were more likely to have a greater risk of incident CVD independent of diabetic status [17, 18] and incident arterial stiffness and nephric microvascular damage [36]. The TyG index was also associated with an increased risk of symptomatic CAD prevalence [37] and CV events in patients with stable CAD [19], NSTE-ACS [38], STEMI undergoing PCI [39], and ACS and DM undergoing PCI [40]. Another study found there was linearity when using TyG as an indicator of ischemic stroke [41]. Our study provided additional information to support previous studies, suggesting the clinical significance of the TyG index for predicting the risk of cardiovascular and cerebrovascular events.

The potential mechanism for interpreting the results of the study was IR. The TyG index has a moderate relationship (r = −0.681) with M rates as measured by the hyperinsulinemic-euglycemic clamp test, suggesting that it may help to identify patients with decreased insulin sensitivity [15]. A prior study demonstrated that the TyG index is closely correlated with insulin-mediated glucose uptake determined by steady-state plasma glucose in nondiabetic patients [42]. In addition, the TyG index demonstrated a good ability to distinguish patients with IR determined by HOMA-IR (AUC = 0.709 in men and 0.711 in women), which suggests that the TyG index may be a good marker for the early identification of IR [43]. Furthermore, another study also showed that the TyG index is a valuable marker for identifying patients with a high risk of diabetes, even better than conventional parameters, such as FPG and TG, in patients with normal FPG [44, 45]. A systematic review assessing the diagnostic accuracy of the TyG index for IR showed that the highest achieved sensitivity was 96% using the HIEC as reference test, while the highest specificity was 99% using HOMA-IR, with a cut-off value of 4.68[Area Under The Curve (AUC) values: 0.59 to 0.88] [46]. Compared with TG/HDL-C, which are other indicators of IR, the TyG index showed a similar AUC to distinguish patients with IR (0.693 and 0.688 for TG/HDL-C and the TyG index, respectively) and a similar ability to predict vascular atherosclerosis defined by a carotid intima-media thickness (IMT) > 0.9 mm [47]. A study in Korean adults indicated that the TyG index is more independently associated with increased arterial stiffness than HOMA-IR [48].

Insulin resistance can be characterized by hyperglycemia with hyperinsulinemia or normoglycemia, which made discordance possible. Previous studies have found that patients with higher glucose and insulin levels might have higher arterial stiffness and concentric remodeling of the heart [49]. Insulin plays a key role in regulating cellular metabolism, and insulin resistance leads to several metabolic changes that can induce the development of cardiovascular disease, such as imbalances in glucose and lipid metabolism. Glucometabolic disorder-caused chronic hyperglycemia triggers oxidative stress and an inflammatory response, finally resulting in cell damage. Lipid metabolism disorders lead to the development of dyslipidemia including high levels of plasma triglycerides, low levels of high-density lipoprotein, and the appearance of small dense low-density lipoproteins. Endothelial dysfunction and dyslipidemia contribute to atherosclerotic plaque formation [50]. A prior study indicated that insulin resistance and inflammatory cytokine interleukins (ILs) contribute to increased TG, HDL-C and matrix-metalloproteinases (MMPs), which trigger structural and functional changes in the CV system [51].

In this study, we selected discordance analyses to compare the capacity to predict the risk between the TyG index and HbA1C or FPG. The C-statistic has been widely used in diagnostic tests and can discriminate diseased patients through sensitivity and specificity. However, when evaluating the models used to predict risks, the C-statistic may not be optimal and may mistakenly exclude important risk predictors. Adding new risk factors may make the risk model more accurate, but the change in the C-statistic is small [52]. Discordance analysis focused on clinical consequences, but not the prediction accuracy which is a focus of conventional standard tests, is a novel approach that discriminate additional positive cases. Discordance analyses could help us understood the consequences of the TyG index via the disagreements between the TyG index and FPG or HbA1C. If the additional cases detected by the new test are more severe and the benefits from treatment are greater than those missed, then the new test will provide a net benefit [53]. An additional reason for choosing discordance analyses is that the TyG index has a tight relationship with FPG and HbA1C, which might reduce the effectiveness of prediction using a traditional comparison approach.

There are several limitations of this study that should be considered. First, there are always residual confounding factors that affect the final results of observational studies. Second, we could not perform a time-dependent analysis because FPG and TG change over time and we only collected a baseline value. Third, the results may be biased because we did not consider the type, intensity and changes in antidiabetic and lipid-lowering treatments, although we adjusted for the use of those medications. Finally, the present results may not be generalizable to other ethnic groups because the participants in our study were only Chinese.

## Conclusions

Patients with a discordantly high TyG index had a significantly greater risk of cardiovascular events regardless of diabetes status. The TyG index, which is easily available with no extra cost, might be a better predictor of cardiovascular risk than FPG or HbA1C. This discordance may support better cardiovascular risk management regardless of diabetes status. However, the best cut-off value and approach for using the TyG index as a target requires further research in future studies.

## Supplementary information

**Additional file 1: Table S1.** Characteristics of the lost participants and eligible participants.

## Data Availability

The datasets used during the current study are available from the corresponding author on reasonable request.

## Abbreviations

BMI: Body mass index
SBP: Systolic blood pressure
MI: Myocardial infarction
PCI: Percutaneous coronary intervention
CABG: Coronary artery bypass grafting
HOMA-IR: Homeostatic model assessment of insulin resistance
FPG: Fasting plasma glucose
HbA1C: Glycosylated hemoglobin
TC: Total cholesterol
HDL-C: High-density lipoprotein-cholesterol
LDL-C: Low-density lipoprotein-cholesterol
TyG index: Triglyceride glucose index
ACS: Acute coronary syndrome
NSTEMI: Non ST-segment elevation myocardial infarction
STEMI: ST-segment elevation myocardial infarction
ACEI: Angiotensin converting enzyme inhibitor
ARB: Angiotensin II receptor blocker
CTO: Chronic total occlusion
LM: Left main artery
LAD: Left anterior descending artery
LCX: Left circumflex artery
RCA: Right coronary artery
DCB: Drug-coated balloon
FFR: Fractional flow reserve
IVUS: Intravascular ultrasound
OCT: Optical coherence tomography
HR: Hazard ratio
CI: Confidence interval
