# Supplementary material for: Discordance between the triglyceride glucose index and fasting plasma glucose or HbA1C in patients with acute coronary syndrome undergoing percutaneous coronary intervention predicts cardiovascular events: a cohort study from China
Source: Cardiovasc Diabetol. 2020 Jul 23;19:116. doi: 10.1186/s12933-020-01091-8 (PMC7379768; doi:10.1186/s12933-020-01091-8)
Supplement: Supplementary file 1 — Additional file 1: Table S1. Characteristics of the lost participants and eligible participants. [file 12933_2020_1091_MOESM1_ESM.doc]

Table S1. Characteristics of the lost participants and eligible participants

|  | Lost participants | | Eligible participants | | *P value** |
| --- | --- | --- | --- | --- | --- |
| N | 1812 | | 9285 | | - |
| Age, y | 60.2±9.92 | | 59.9±10.05 | | 0.185 |
| Male,n (%) | 1366(75.4) | | 6997(75.4) | | 0.98 |
| BMI,kg/m2 | 26.6±12.76 | | 26.2±9.21 | | 0.168 |
| Heart rate,bpm | 71±12.9 | | 72±12.72 | | 0.003 |
| SBP, mmHg | 127.3±23.18 | | 128.6±20.86 | | 0.024 |
| Medical history and risk factors, n (%) | |  | |  | |
| Current smoker | 680(37.5) | | 3499(37.9) | | 0.74 |
| Hypertension | 1164(64.2) | | 6104(65.7) | | 0.219 |
| Diabetes | 634(35) | | 3423(36.9) | | 0.129 |
| Dyslipidaemia | 1419(78.3) | | 7131(76.8) | | 0.162 |
| Previous MI | 238(13.1) | | 1156(12.5) | | 0.421 |
| Previous Stroke | 94(5.2) | | 485(5.2) | | 0.95 |
| Previous PCI | 451(24.9) | | 2264(24.4) | | 0.647 |
| Previous CABG | 48(2.6) | | 220(2.4) | | 0.478 |

Values are mean± SD, median (interquartile range), or n (%). *p value for test of difference across the 2 lost participant and eligible participant groups by the chi-square test for categorical variables or analysis of variance for continuous variables or Kruskal-Wallis test for nonparametric comparisons. BMI body mass index, SBP systolic blood pressure, MI myocardial infarction, PCI percutaneous coronary intervention, CABG Coronary Artery Bypass Grafting.
